# A metabolome-wide case-control study of african american breast cancer patients

**DOI:** 10.1186/s12885-023-10656-1

**Published:** 2023-02-23

**Authors:** Jiajun Luo, Muhammad G. Kibriya, Hui Chen, Karen Kim, Habibul Ahsan, Olufunmilayo I. Olopade, Christopher S. Olopade, Briseis Aschebrook-Kilfoy, Dezheng Huo

**Affiliations:** 1 Department of Public Health Sciences, University of Chicago Biological Sciences, 5841 S. Maryland Ave. MC2000, 60637 Chicago, IL USA; 2 Institute for Population and Precision Health, University of Chicago, Chicago, IL USA; 3 Comprehensive Cancer Center, University of Chicago, Chicago, IL USA; 4 Mass Spectrometry Core, University of Illinois at Chicago, Chicago, IL USA; 5 Department of Medicine, University of Chicago, Chicago, IL USA

**Keywords:** breast cancer, metabolome, metabolomics, prostaglandin, leukotriene, glycerophospholipid

## Abstract

**Background:**

Breast cancer survivors face long-term sequelae compared to the general population, suggesting altered metabolic profiles after breast cancer. We used metabolomics approaches to investigate the metabolic differences between breast cancer patients and women in the general population, aiming to elaborate metabolic changes among breast cancer patients and identify potential targets for clinical interventions to mitigate long-term sequelae.

**Methods:**

Serum samples were retrieved from 125 breast cancer cases recruited from the Chicago Multiethnic Epidemiologic Breast Cancer Cohort (ChiMEC), and 125 healthy controls selected from Chicago Multiethnic Prevention and Surveillance Study (COMPASS). We used liquid chromatography-high resolution mass spectrometry to obtain untargeted metabolic profiles and partial least squares discriminant analysis (PLS-DA) combined with fold change to select metabolic features associated with breast cancer. Pathway analyses were conducted using Mummichog to identify differentially enriched metabolic pathways among cancer patients. As potential confounders we included age, marital status, tobacco smoking, alcohol drinking, type 2 diabetes, and area deprivation index in our model. Random effects of residence for intercept was also included in the model. We further conducted subgroup analysis by treatment timing (chemotherapy/radiotherapy/surgery), lymph node status, and cancer stages.

**Results:**

The entire study participants were African American. The average ages were 57.1 for cases and 58.0 for controls. We extracted 15,829 features in total, among which 507 features were eventually selected by our criteria. Pathway enrichment analysis of these 507 features identified three differentially enriched metabolic pathways related to prostaglandin, leukotriene, and glycerophospholipid. The three pathways demonstrated inconsistent patterns. Metabolic features in the prostaglandin and leukotriene pathways exhibited increased abundances among cancer patients. In contrast, metabolic intensity in the glycerolphospholipid pathway was deregulated among cancer patients. Subgroup analysis yielded consistent results. However, changes in these pathways were strengthened when only using cases with positive lymph nodes, and attenuated when only using cases with stage I disease.

**Conclusion:**

Breast cancer in African American women is associated with increase in serum metabolites involved in prostaglandin and leukotriene pathways, but with decrease in serum metabolites in glycerolphospholipid pathway. Positive lymph nodes and advanced cancer stage may strengthen changes in these pathways.

**Supplementary Information:**

The online version contains supplementary material available at 10.1186/s12885-023-10656-1.

## Introduction

Breast cancer survivorship has been improved since 1990s due to advances in early detection and cancer therapy [1]. Over 90% of all female breast cancer patients survived 5 years after their initial diagnosis [2]. Currently, more than 3.8 million women in the US are estimated to have a history of breast cancer, comprising the largest group of cancer survivors [1]. Meanwhile, breast cancer patients generally report poorer health compared to the general population even after successful treatment [3, 4]. Higher risks for cardiovascular disease (CVD) [5–7], pulmonary disease [8], fatigue [9], chronic pain [10], and cognitive decline [11] are documented among breast cancer survivors. Studies have suggested that CVD is the largest single cause of death among breast cancer survivors, exceeding cancer-related causes [12].

The long-term sequelae of breast cancer are usually attributed to the side effects of cancer therapies, because radiotherapy, chemotherapy, and trastuzumab therapy are able to induce cardiotoxicity and have been linked to higher risks for cardiovascular and pulmonary diseases [13–23]. However, not all sequelae can be explained by cancer therapies. Genetic susceptibility [24, 25] and shared risk factors including age, obesity, and physical inactivity [26–30], also contribute to the development of long-term sequelae after breast cancer diagnosis. Among all potential risk factors, biological changes induced by breast cancer among survivors should not be overlooked.

Given that these long-term sequelae appear to manifest nearly 5 years after initial diagnosis of breast cancer [5, 7], there exists a potential window period for interventions mitigating disease burdens. Meanwhile, minority groups such as African American face a disproportionately large burden of both breast cancer and chronic diseases [31]. Therefore, understanding the cause and etiology of these sequelae among breast cancer patients will be crucial for the development of such medical interventions, especially among minority groups.

As disturbances in metabolic activities underlie most diseases, the study of the metabolome can provide important insight into the etiology of breast cancer sequelae as well as offer the potential to identify metabolic pathways for clinical interventions [32]. More recently, metabolomics technologies give us the ability to measure thousands of metabolites in biological samples, assisting researchers in the investigation of metabolic changes. Metabolomics has demonstrated an emerging and promising role in the diagnosis and prognosis of chronic diseases and clinical interventions [33].

Within this context, we aim to investigate the metabolic profile of breast cancer patients soon after diagnosis and identify potential biological pathways using the state-of-the-art metabolomics technologies among a case-control study with 125 breast cancer cases and 125 controls that were frequency-matched by age. All the participants were African American women residing in Chicago, offering the opportunity to mitigate the larger burdens of both breast cancer and chronic disease in this group [31].

## Method

### Study population

The Chicago Multiethnic Epidemiologic Breast Cancer Cohort (ChiMEC) was initiated as a hospital-based case-control study to facilitate research on the effects of high-penetrance susceptibility genes, common genetic variants, and environmental risk factors for breast cancer [34–37]. Breast cancer cases were followed for survival, disease recurrence, and other outcomes to form the ChiMEC cohort, with a current sample size of 5097 patients [38]. Patients diagnosed or treated at the University of Chicago Hospitals were ascertained through the cancer risk clinic and breast center. Clinical, pathological, and treatment data were collected via electronic medical records. Epidemiological risk factor data were collected via a questionnaire. A biobank was established, collecting blood and tumor samples. This analysis randomly selected female African American patients who were ≥ 18 years of age at diagnosis, lived in Chicago, enrolled in the ChiMEC study between 2012 and 2018, had histologically diagnosed non-metastatic invasive breast cancer, and had serum samples available. Stage IV distant metastatic patients were excluded as these patients might have tumor cells in circulation and experience dramatic metabolic changes.

The healthy controls were selected from the Chicago Multiethnic Prevention and Surveillance Study (COMPASS), a large scale, longitudinal cohort study with a current sample size of 7728 participants from 72 of the 77 Chicago community areas [39]. Residents of the greater Chicago area were eligible for COMPASS if they were: (1) 18 or older at the time of enrollment; (2) able to give consent and provide survey data in English or Spanish; (3) willing to provide blood, urine, saliva samples, and access to medical records. Recruitment strategies to increase minority enrollment have included a predominantly minority interviewer team and focus on recruitment in census tracts with minority and diverse populations as the primary sampling unit. Enrollment entails the completion of a 1-hour long survey, consenting for past and future medical records from all sources, the collection of clinical and physical measurement data and the on-site collection of biological samples including blood, urine and saliva. On collection, all biological samples are processed and liquated within 24 h before long-term storage and subsequent analysis. Participants completed an extensive survey providing information on medical history, socioeconomic status, psychosocial variables, lifestyle behaviors, social environment, immune status, use of medical services, medication use, among other covariates. Blood collection occurred at the same time as consent and the in-person interview.

#### Informed consent

has been obtained from all ChiMEC and COMPASS participants. For the current analysis, 125 African American participants were randomly selected from ChiMEC and COMPASS, respectively, frequency-matched by age. In addition to data collection through questionnaire interview and electronic medical records, we also conducted geocoding analysis based on residence addresses of participants in both studies to collect neighborhood-level characteristics, and we calculated area deprivation index (ADI) [40].

### High-resolution metabolomics

The precipitation of proteins were performed using the Ostro 96-well plate by following the manufacturer’s protocol for each serum sample. In brief, 100uL of serum were placed in the well with 100uL of surrogate standard Loratadine. Then 200uL of cold solvent (acetonitrile:formic acid 99:1) were added, followed by gentle mixing before the filtration by manifold processor. The procedure was repeated with 400ul cold solvent (acetonitrile/water/formic acid 3:1:1%). The resulting solution were dried by nitrogen flow and reconstituted in 200uL solvent (acetonitrile/water 1:1) with spiked-in internal standards (Celecobix and 4-Aminobiphenyl).

For testing the serum extraction procedure, a quality control sample was prepared by mixing same volumes of all serum samples. The same extraction protocol was performed on these quality control samples randomly placed in the well plate. The quality control extracts together with the extraction blanks were injected during the sample analysis.

Liquid chromatography mass spectrometry (LC-MS) analysis of the metabolite extracts was performed using an Agilent 6545 Q-TOF and 1290UPLC system controlled by the Agilent Mass Hunter acquisition software. The mass spectrometer was operated in 2 GHz extended dynamic range mode employing precursor ion analysis for relative quantification experiments in positive/negative ion modes. Internal references were used for calibration. Mobile phase A was 0.1% formic acid in H2O and B was 0.1% formic acid in acetonitrile. The samples were loaded onto a 2.1 × 100 mm Agilent Poroshell C18, 1.9 μm column (Agilent Technologies Inc., Santa Clara, CA, USA) and separation performed using an Agilent 1290 Ultra Performance Liquid Chromatography system at a flow rate of 300 µL/ min. The gradient started with 2%B and was increased to 65% in 11 min, then to 95%B in 2 min. The gradient was held at 95%B for 4 min. A post column equilibration time of 4 min was used for all runs. The post time was set to 3 min to re-equilibrate after each run. Source parameters were as follows: gas temp (300 °C), drying gas (11 L/min), nebulizer (35 psi), sheath gas temp (350 °C), sheath gas flow (12 L/min ), VCap (3000 V), and fragmentor (145 V). The column compartment was held at 35 °C. Data was collected for relative quantification using a scan speed of 3 MS spectra per second. Pooled quality control samples were injected across every 10 sample injections. Each extract was separated and quantitated only once.

The surrogate standard was used to track the recovery after extraction and reconstitution. Each sample was used within ± 15% recovery. This protocols were widely used in metabolomics studies [41–43]. As suggested by the literature, the protocols used in this study offered the greatest extraction performance, though the survivability of certain metabolites cannot be evaluated [41].

### Statistical analysis

Metabolic features that were present in at least 80% of one group and > 50% of all samples were filtered and maintained in following analyses. After filtering, missing values in these features were imputed by one-half of the lowest signal detected for that feature across all samples as suggested by prior studies [44]. Log2-transformed feature intensities were used for analyses. To control for confounding from different aspects, we used residuals of intensities derived from linear mixed effects regression models against potential confounders including age, marital status, tobacco smoking, alcohol drinking, type 2 diabetes, and area deprivation index of residential address [40]. To further eliminate potential confounding arising from spatial variations, we included residential zipcode as random intercepts in the mixed effects models.

Partial least squares discriminative analysis (PLS-DA) was employed to identify differential features between cases and controls. PLS-DA is a supervised multivariate analytical approach for dimensionality reduction that maximizes covariance between cancer status and feature intensities [45]. A Variable Importance in Projection (VIP) score is generated for each feature in the PLS-DA model. The VIP score estimates the importance of each feature in the model and is commonly used for feature selection. Features with a VIP score > 2 in PLS-DA and fold change (FC) > 2 were considered clinically importance. Moreover, to evaluate the performance of selected features, we conducted 10-fold cross-validation tests utilizing the support vector machine and calculated the classification accuracy of the selected features. All feature selection approaches were implemented with the R package mixOmics v6.3.1.

### Pathway analysis

The enriched metabolic pathways differential between controls and cases were identified using Mummichog (v. 1.0.10) [46]. All features were included in the pathway enrichment analysis, while the selected features were denoted as significant. Mummichog is a novel and reliable algorithm for pathway enrichment analysis designed specifically for high-resolution LC-MS. This algorithm has been proven to be valid and reflect real biological activity [47–50]. To reduce false positive match rate, the Mummichog algorithm requires all annotated metabolites to present in at least their primary adduct (M + H or M-H for positive and negative mode, respectively). We used a P value of 0.05 as the threshold. Only enriched pathways with at least 3 overlapping metabolites were kept for further interpretation.

### Subgroup analysis

Clinical characteristics related to breast cancer had the potential to modify metabolic activities among patients. To evaluate the influence of these variables, we conducted subgroup analysis based on selected clinic characteristics. Specifically, we repeated aforementioned metabolomics analysis using cases stratified by treatment, lymph node, and cancer stage. To eliminate the influence of cancer treatment on metabolomics in breast cancer cases, we included only cases without any treatment before serum collection in the analysis. To examine the impact of cancer extent on metabolomics, we also conducted subgroup analysis based on cancer stage and lymph node. In summary, we have a total of seven scenarios in the analysis of this study: (1) all breast cancer cases as described above; (2) cases without any cancer treatment (chemotherapy, radiotherapy, and surgery) before serum collection; (3) cases with one of treatment modalities (chemotherapy, radiotherapy, and surgery) before serum collection; (4) cases with negative lymph nodes; (5) cases with positive lymph nodes; (6) cases with stage I cancer; and (7) cases with stages II or III cancer.

Study protocols of both studies (ChiMEC and COMPASS) were approved by the Institutional Review Board at the University of Chicago. All participant recruitment, human sample experiment, and data analysis in this study were carried out in accordance with Helsinki Declaration guidelines.

## Results

The average age was 57.1 years for controls and 58.0 years for cases (Table 1). There were more current tobacco smokers (40.8% vs. 13.6%) and alcohol drinkers (61.6% vs. 44.8%) among controls compared to cases. The prevalence of type 2 diabetes were comparable between these two groups (13.6% vs. 11.2%). Both cases and controls lived in similar communities, as indicated by zip codes, in south side of Chicago. The areas that cases and controls lived had similar ranks of area deprivation index (median value: 71 vs. 66). Compared to all ChiMEC population [38], the breast cancer cases in this study demonstrated consistent distributions of demographic characteristics because of the random selection process, as among the original ChiMEC African American patients the average age was 59.6 years and 15.8% reported current smoking. Similarly, the demographic characteristics of healthy controls in this study also exhibited no substantial difference from the original COMPASS cohort, as the average age was 53.7 years, 47.8% reported current smoking, and 10.1% had type 2 diabetes in the original population.

**Table 1:** Distribution of selected characteristics among participants

	Controls (n = 125)	Cases (n = 125)
**Characteristics**		
Age at enrollment, mean ± SD	57.1 ± 13.8	58.0 ± 13.9
Marital status, n (%)		
Single	63 (50.4)	61 (48.8)
Married or partnered	18 (14.4)	34 (27.2)
Divorced or widowed	44 (35.2)	30 (24.0)
Current tobacco smoker, n (%)		
No	74 (59.2)	108 (86.4)
Yes	51 (40.8)	17 (13.6)
Current alcohol drinker, n (%)		
No	48 (38.4)	69 (55.2)
Yes	77 (61.6)	56 (44.8)
Type 2 diabetes, n (%)		
No	108 (86.4)	111 (88.8)
Yes	17 (13.6)	14 (11.2)
Area Deprivation Index national rank, Median (interquartile range)	71 (57–85)	66 (49–80)
**Cancer-related characteristics**		
Cancer stage group		
I		49 (39.2)
II		58 (46.4)
III		18 (14.4)
Lymph node involvement		
Negative		72 (57.6)
1–3 node positive		45 (36.0)
4 + node positive		7 (4.8)
Missing		1 (0.8)
Surgery, n (%)		
No		3 (2.4)
Yes, after serum collection		97 (77.6)
Yes, before serum collection		25 (20.0)
Chemotherapy, n (%)		
No		47 (37.6)
Yes, after serum collection		32 (25.6)
Yes, before serum collection		46 (36.8)
Radiotherapy, n (%)		
No		43 (34.4)
Yes, after serum collection		73 (58.4)
Yes, before serum collection		9 (7.2)
Any treatment before serum collection, n (%)		
No		68 (54.4)
Yes		57 (45.6)

Among breast cancer patients in this study, the median time from diagnosis to serum collection was 72 days. There were 49, 58, and 18 patients having breast stage I, II, and III breast cancer, respectively. More than half cancer patients had lymph node tumor negative disease (57.6%). Almost all patients received breast surgery (mastectomy or lumpectomy), and 25 of them received surgery before serum collection (median 98 days). Most patients (62.4%) received chemotherapy, and 46 of them received chemotherapy before serum collection (median 158 days after initiation of chemotherapy). There were 65.6% receiving radiotherapy, and only 9 of them before serum collection. There were 57 (45.6%) patients receiving any treatment (chemotherapy, radiotherapy, and surgery) before serum collection, while the rest 68 patients had sera collected after date of diagnosis and before any cancer treatment.

**Fig. 1:**
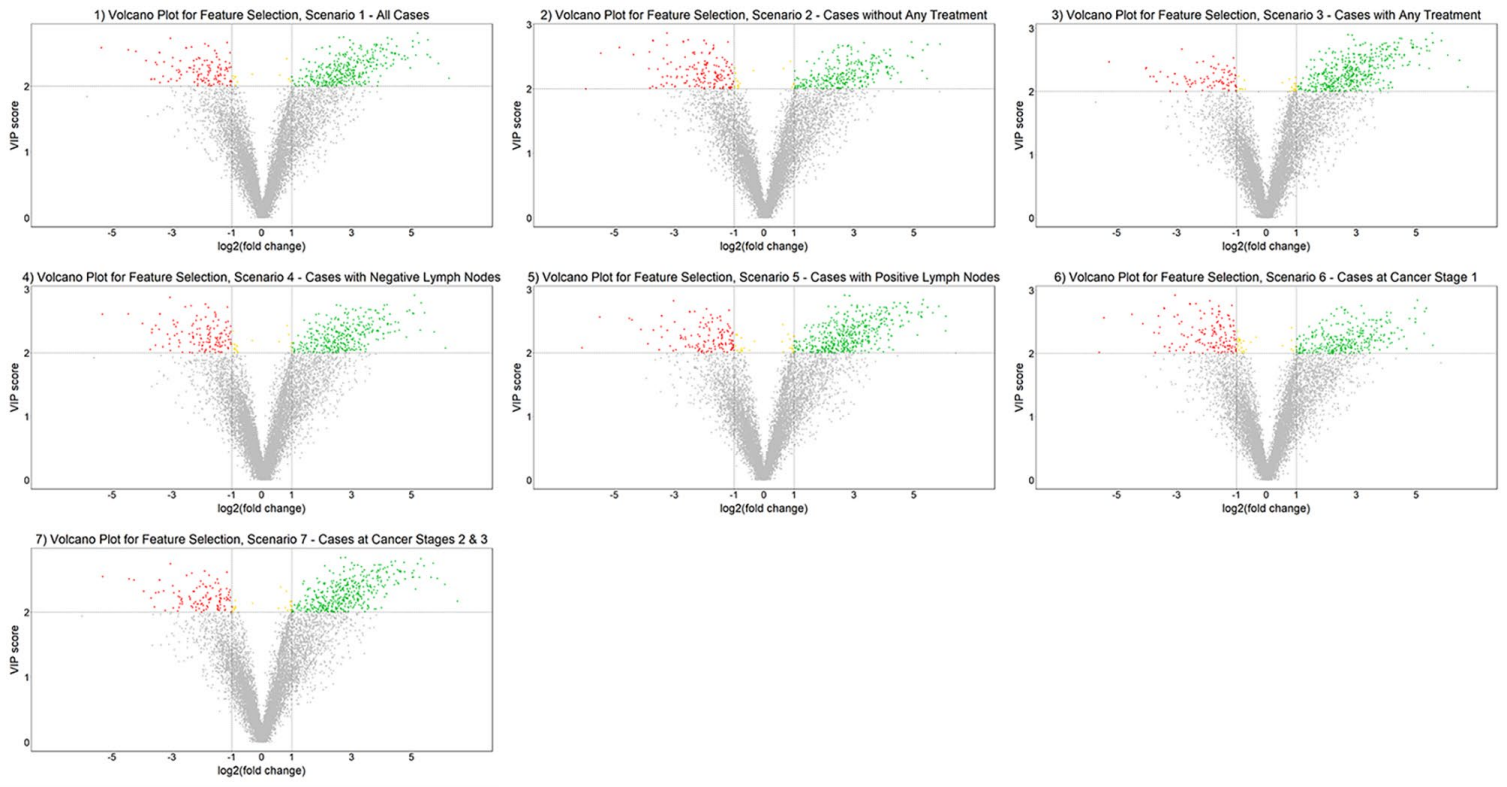
Identification of metabolic features associated with breast cancer. The positive fold change (log2) indicates higher feature intensity among cases. (1) Scenario 1, all breast cancer cases (n = 125); (2) Scenario 2, cases without any treatments (chemotherapy, radiotherapy, and surgery) before serum collection (n = 68); (3) Scenario 3, cases with any treatments (chemotherapy, radiotherapy, and surgery) before serum collection (n = 57); (4) Scenario 4, cases with negative lymph nodes (n = 72); (5) Scenario 5, cases with positive lymph nodes (n = 52); (6) Scenario 6, cases with stage I cancer (n = 49); (7) Scenario 7, cases with stage II/III cancer (n = 76)

In total, we detected 15,829 features, among which 12,023 remained after filtering for missing values. When we compared controls with all cases, 518 unique metabolic features were selected using PLS-DA after adjusting for aforementioned confounders, while setting the VIP scores > 2 (Table 2; Fig. 1, Scenario 1). The classification error rate obtained from the 10-fold cross-validation was 4.8%, suggesting the set of discriminatory features effectively separate the cases and controls. Among the 518 features, 507 (97.9%) features had FC > 2 and were eventually used for enriched pathway. In our subgroup analysis, the classification error rates ranged from 2.9 to 8.4%, and the final numbers of selected metabolic features from 478 to 612. The scenario where breast cancer cases had positive lymph node had the lowest classification error rate and the largest number of selected metabolic features. In contrast, the scenario where cases with stage I cancer had the smallest number of selected metabolic features. See supplemental **Table S1** for VIP scores and FC of each feature in each scenario in this study.

**Table 2:** Model performance for feature selection

Overall and subgroup analysis (number of cancer cases)*	Number of features with VIP score > 2 in PLS-DA	Classification error rate of PLS-DA	Final number of features with FC > 2
Scenario 1: All cases (n = 125)	518	4.8%	507
Scenario 2: Cases without any treatment before serum collection (n = 68)	477	5.2%	457
Scenario 3: Cases with some treatment before serum collection (n = 57)	602	8.4%	573
Scenario 4: Cases with negative lymph nodes (n = 72)	506	8.4%	485
Scenario 5: Cases with positive lymph nodes (n = 52)	644	2.9%	612
Scenario 6: Cases with stage I cancer (n = 49)	512	7.2%	478
Scenario 7: Cases with stage II or III cancer (n = 76)	584	5.6%	560

FC, fold change; PLS-DA, partial least square discriminatory analysis; VIP, variable importance in projection

*The same 125 healthy controls were compared with

We evaluated whether metabolic features selected in this study were enriched in specific metabolic pathways using Mummichog (Table 3). When using all breast cancer cases (Scenario 1), enriched pathway analysis indicated that three metabolic pathways were differentially enriched between cases and controls with a P-value < 0.05 and had at least 3 overlapped metabolites when we compared breast cancer cases with healthy controls. The three metabolic pathways were prostaglandin formation from arachidonate (8 overlap metabolites), leukotriene metabolism (8 overlap metabolites), and glycerophospholipid metabolism (6 overlap metabolites). The first two pathways, prostaglandin formation from arachidonate and leukotriene metabolism, indicate an inflammatory response. The last pathways is lipid-related metabolic pathway, which is closely related to essential biological structures and functions. Tentative annotation results of metabolites in each differentially enriched pathways and FCs of these features are present in Table 4. Notably, the three pathways demonstrated different patterns. In prostaglandin and leukotriene pathways, FCs of all significant metabolic features were positive, meaning that these features in the two pathways exhibited increased abundance among breast cancer patients. In contrast, in the glycerophospholipid pathway, all significant metabolic features had negative FC and thus showed a decreased intensity among breast cancer patients.

**Table 3:** Enriched metabolic pathways associated with breast cancer status

Pathway	Pathway size^a^	Scenario 1^b^		Scenario 2^b^		Scenario 3^b^		Scenario 4^b^		Scenario 5^b^		Scenario 6^b^		Scenario 7^b^	
		Overlap size^c^	P value^d^	Overlap size^c^	P value^d^	Overlap size^c^	P value^d^	Overlap size^c^	P value^d^	Overlap size^c^	P value^d^	Overlap size^c^	P value^d^	Overlap size^c^	P value^d^
Prostaglandin formation from arachidonate	58	8	0.018	7	0.025	9	0.008	7	0.030	10	0.003	6	0.046	9	0.010
Leukotriene metabolism	56	8	0.020	6	0.076	9	0.011	7	0.037	9	0.010	5	0.122	9	0.014
Glycerophospholipid metabolism	48	6	0.040	6	0.025	7	0.016	7	0.009	7	0.015	5	0.052	7	0.019

^a^ The pathway size is the number of detected empirical compounds for each pathway

^b^ In scenarios 1 to 7, the cases used to compare healthy controls were: (1) all breast cancer cases, (2) cases without any treatments (chemotherapy, radiotherapy, and surgery) before serum collection, (3) cases with some treatments (chemotherapy, radiotherapy, and surgery) before serum collection, (4) cases with negative lymph nodes, (5) cases with positive lymph nodes, (6) cases with stage I cancer, and (7) cases with stage II/III breast cancer, respectively

^c^ The overlap size is the number of significant empirical compounds

^d^ P values calculated by Mummichog were gamma-adjusted P values based on permutation tests by resampling from the reference list

**Table 4:** Tentative match of the serum metabolic features associated with breast cancer to the metabolites within mummichog enriched pathways

Pathway	m/z	RT (s)	Tentative match	Fold change (log2)^a^						
				Scenario 1^b^	Scenario 2^b^	Scenario 3^b^	Scenario 4^b^	Scenario 5^b^	Scenario 6^b^	Scenario 7^b^
**Prostaglandin formation from arachidonate**	410.2634	12.13	12-hydroperoxyeicosatetraenoate glyceryl ester	3.18	2.94	3.47	3.03	3.37	2.94	3.34
	424.2432	12.27	15-oxo-Prostaglandin E2 glyceryl ester	2.78	2.31^c^	3.35	2.43	3.29	2.33^c^	3.08
	368.2161	10.16	20-dihydroxyleukotriene B4	2.33	2.12	2.57	2.13	2.61	2.27	2.36
	368.2166	10.22	Prostaglandin G2	2.17	2.07	2.28	2.07	2.31	2.21	2.14
	366.2008	10.24	11-dehydro-15-keto-TXB2	3.24	3.19	3.30	3.22	3.26	3.13	3.32
	352.2236	10.93	Prostaglandin E2	2.19	1.99	2.42	1.94^c^	2.53	1.88^c^	2.39
	424.2438	12.60	15-oxo-Prostaglandin E2 glyceryl ester	2.62	2.19	3.14	2.37	3.00	2.21	2.89
	370.2706	11.99	Anandamide	4.18	3.66	4.79	3.88	4.56	3.78	4.43
	366.2008	10.20	12-oxo-20-dihydroxy-leukotriene B4	3.24^c^	3.19^c^	3.30	3.22^c^	3.26	3.13^c^	3.32
	368.2161	10.16	20-dihydroxyleukotriene B4	2.33^c^	2.12^c^	2.57^c^	2.13^c^	2.61	2.27^c^	2.36^c^
**Leukotriene metabolism**	364.1858	12.20	20-carboxy-leukotriene-B4	2.30	2.09	2.54	2.19	2.43	2.16	2.38
	368.2161	10.16	20-dihydroxyleukotriene B4	2.33	2.12	2.57	2.13	2.61	2.27	2.36
	496.2647	10.87	Leukotriene D4	2.06	1.92	2.23	2.09	1.99	2.06	2.06
	382.1958	10.76	12-oxo-20-trihydroxy-leukotriene B4	3.27	2.89^c^	3.72	3.10	3.51	3.06^c^	3.40
	366.2008	10.24	20-COOH-10,11-dihydro-LTB4	3.24	3.19	3.30	3.22	3.26	3.13^c^	3.32
	370.1772	9.29	12,20-dioxo-leukotriene B4	3.20	2.94	3.51	2.91	3.59	2.89	3.40
	352.2236	10.93	Prostaglandin E2	2.19	1.99^c^	2.42	1.94^c^	2.53	1.88^c^	2.39
	366.2008	10.24	11-dihydro-LTB4	3.24	3.19	3.30	3.22	3.26	3.13	3.32
	630.4014	13.93	Kurilensoside F	1.18^c^	0.97^c^	1.43	1.29^c^	1.05	1.21^c^	1.16
**Glycerophospholipid metabolism**	299.2820	14.07	Sphingosine	-1.14	-1.22	-1.05	-1.13	-1.16	-1.18	-1.12
	687.4946	14.68	Diacylglycerol	-1.91	-2.09	-1.69	-1.87	-1.96	-1.81	-1.96
	278.2252	16.23	(6Z,9Z,12Z)-Octadecatrienoic acid	-2.09	-2.26	-1.89	-2.17	-1.96	-2.18	-2.03
	301.2975	11.47	Sphinganine	-1.30	-1.38	-1.20	-1.34	-1.23	-1.32^c^	-1.28
	280.2400	15.23	Linoleic acid (all cis C18:2) n-6	-1.72	-1.92	-1.48	-1.75	-1.68	-1.74	-1.71
	299.2822	11.17	3-dehydrosphinganine	-2.26	-2.39	-2.09	-2.29	-2.19	-2.25	-2.26
	417.2354	11.59	1-(1-Alkenyl)-sn-glycero-3-phosphate	-1.67^c^	-1.40^c^	-2.00	-1.50	-1.91	-1.57^c^	-1.74

^a^ A fold change larger than 0 indicated higher intensity among breast cancer cases than controls

^b^ In scenarios 1 to 7, the cases used to compare healthy controls were: (1) all breast cancer cases, (2) cases without any treatments (chemotherapy, radiotherapy, and surgery) before serum collection, (3) cases with some treatments (chemotherapy, radiotherapy, and surgery) before serum collection, (4) cases with negative lymph nodes, (5) cases with positive lymph nodes, (6) cases with stage I cancer, and (7) cases with stage II/III breast cancer, respectively

^c^ The metabolite was not statistically significant in this pathway under the scenario

The association directions of features in the three pathways were all consistent across all scenarios (Table 4). In the subgroup analysis using data from cases with sera collected after breast diagnosis and before any cancer treatment, we found that the three pathways were still significant or marginal significant (Table 3, Scenario 2). The directions and FC magnitudes were also consistent in overall and the subgroup analysis (Table 4, Scenarios 1 and 2). Interestingly, we found slightly larger number of overlap metabolic features (Table 3) and generally larger effect sizes (Table 4) in prostaglandin and leukotriene pathways when comparing the subgroup analysis of lymph node positive cases (Scenarios 5) to the subgroup analysis of lymph node negative cases (Scenarios 4), while there were similar effect sizes in the glycerophospholipid pathway between the two scenarios. Similarly results were found when comparing the subgroup analysis of stage II/III cancer cases (Tables 3 and 4, Scenario 7) to the subgroup analysis of stage I cancer cases (Scenarios 6). Except for the aforementioned three metabolic pathways, no other significant pathways were observed in the subgroup analyses.

## Discussion

In this study, we used untargeted high-resolution metabolomics approach to investigate the metabolic changes soon after diagnosis among breast cancer patients compared with healthy controls. With more than 15,000 metabolic features detected from serum of the study samples, we found that metabolic pathways related to inflammatory reactions and lipid metabolism were differentially enriched among breast cancer patients, providing clues for the long-term sequelae manifested after breast cancer diagnosis. Specifically, two inflammatory reactions pathways, prostaglandin formation from arachidonate and leukotriene metabolism, were differentially enriched between controls and cases. Metabolic features in these two pathways showed an increased abundance among breast cancer patients. The changes in these pathways were strengthened when only considering breast cancer cases with positive lymph nodes or stage II/III cancer. By contrast, in the enriched lipid-related pathway, glycerophospholipid metabolism, the intensity of metabolic feature were mainly downregulated among cancer patients.

Glycerophospholipids are the most abundant lipids in virtually all mammalian membranes. Dysfunction in glycerophospholipid metabolism is among the most prominent metabolic alterations in cancer [51], as cancer cells need to continually regulate glycerophospholipids for membrane production, energy acquisition, and molecular signaling [52]. Therefore, it is likely that the observed changes in glycerophospholipid metabolism in this study are the direct consequence of breast cancer and thus suggest disruptions among breast cancer patients. The altered lipid metabolism further disrupts cholesterol homeostasis [53, 54], which has been linked to the progression of various chronic disease [55–58]. Given the crucial role of glycerophospholipid in cell membranes and surfactant, it is natural and intuitive that dysfunction of glycerophospholipid metabolism results in a series of chronic diseases [59]. However, the long-term impact of the disruptions in glycerophospholipid metabolism is under-investigated, warranting more studies.

In addition to serving as key structural components of membranes, glycerophospholipids can also provide arachidonic acid, the precursor of prostaglandins and leukotrienes [60]. As a key inflammatory intermediate, arachidonic acid is released from cell membranes controlled by glycerophospholipids and then converted to eicosanoids through pathways including: the lipoxygenase (LOX) pathway, where arachidonic acids is dioxygenated to produce hydroperoxyeicosatetraenoic acid (HPETE) and then further converted to leukotrienes; and the cyclooxygenase (COX) pathway that produces prostaglandin [61]. Prostaglandins and leukotrienes are two major pro-inflammatory mediators, and their metabolic pathways were observed to be differentially enriched among breast cancer patients in this study.

The most common type of prostaglandins is prostaglandin E_2_ (PGE_2_) whose link to CVD has been extensively documented in the literature [62]. PGE_2_ is involved in a variety of biological functions in the cardiovascular system, including vascular tone regulation, cardiac remodeling, and cardiac inflammation [62]. While clinical studies are still lacking, evidence from animal experiments reveals that PGE_2_ is associated with higher risks of heart failure, reperfusion injury, arrhythmias, hypertension, hypertensive heart disease, and atherosclerosis [62]. In this study, we observed that PGE_2_ was increased among breast cancer patients.

Leukotrienes have been linked to increased risks for CVD and other diseases by prior studies [63]. The overproduction of leukotrienes is a major cause of inflammation [64]. Leukotriene D_4_ (LTD_4_) is one of leukotrienes that retains biological activity. In our study, we observed that LTD_4_ was increased intensity among breast cancer patients, revealing another potential mechanistic pathway of the ling-term sequelae after breast cancer diagnosis. Recently, given the close relationship between CVD and leukotrienes, there is an emerging interest develop cardiovascular drugs based on leukotriene modifiers, which are expected to mitigate stroke, myocardial infarction, and atherosclerosis [65].

In the subgroup analysis, though consistent with our main findings, the changes in these differentially enriched pathways were somewhat strengthened in the scenario where cases had positive lymph nodes or stage II/III cancer, while attenuated in scenarios where cases had negative lymph nodes or stage I cancer. The observation of more extent of cancer with stronger associations with the aforementioned three pathways is reasonable and anticipated, as ancillary lymph nodes are considered the most important route for breast cancer to spread to blood and then other organs.

The study has several strengths. First, to our knowledge, this study is the first one that employ metabolomics approaches to analyze the difference in metabolism between breast cancer cases and healthy controls. Findings from this study identified potential metabolic target for clinical intervention that mitigates long-term sequelae among breast cancer patients. Second, substantial efforts have been made to ensure the homogeneity of the study population with an aim to preclude the influence from external factors. Specifically, the cases and controls were matched by age and all of them were African Americans, so that variations arising from genetics and age could be reduced. Moreover, all participants were recruited from similar communities in south side of Chicago and random effects of spatial variations were further included in the regression model. Therefore, impacts of environmental factors, such as air pollution and neighborhood disadvantages, could be minimized. All these approaches were expected to increases the validity and biological reliability of our results, though we cannot completely rule out external influences.

Our study also has some limitations that warrant caution in interpretation. First, the breast cancer samples are heterogeneous regarding time of serum collection and cancer stage. Chemotherapy and radiotherapy can have short and long-term influence on metabolic profile in circulation, while breast surgery can have short-term impact on inflammatory markers. In the subgroup analysis using samples collected after diagnosis and before any of these treatment, the main study findings remained, suggesting that the differentially regulated pathways identified in this study may be due to intrinsic cancer metabolism. The heterogeneity by cancer stage, as discussed above, suggests that these differentially regulated pathways depend on extent of breast cancer. Second, without further metabolite identification using tandem MS, we could only tentatively annotate the extracted features and enriched pathways using computational approaches. This is an inherent challenge in untargeted metabolomics studies. We could not rule out false matches that influenced our interpretation. Observations from the current study only provide helpful, but not conclusive, suggestions for metabolic changes related to breast cancer. Future studies are warranted to improve the identification of metabolites using either tandem MS or internal standards. Third, the study population were all African Americans, therefore, generalizability to other populations might be weakened. Particularly, genetic variations across racial/ethnic groups could prevent us generalizing conclusions from African Americans to other groups. However, given African American females face higher disease burdens of both breast cancer and chronic diseases, our results can also provide valuable insights regarding public health and clinical interventions.

In summary, we applied high-resolution metabolomics to identify perturbations in the serum metabolome associated with breast cancer. We observed metabolic pathways consistent with inflammatory reactions and lipid metabolism that may contribute to long-term sequelae among breast cancer patients. Particularly, we observed differentially enriched metabolic pathways of prostaglandins and leukotrienes with increased abundances of metabolic features among cancer patients. Changes in these two pathways appear to be strengthened by positive lymph nodes, and weakened at negative lymph nodes. We also observed decreased metabolic intensities in the pathway of glycerolphospholipid among breast cancer patients. The findings from this study may provide insights to identify clinical intervention targets that mitigate long-term sequelae among breast cancer patients.

## Electronic supplementary material

Below is the link to the electronic supplementary material.

Supplementary Material 1

## Data Availability

The data that support the findings of this study are available on request from the corresponding author, D.H. The data are not publicly available due to their containing information that could compromise the privacy of research participants.
